# A Few Surgical Observations

**Published:** 1894-08

**Authors:** J. D. Westervelt

**Affiliations:** Surgeon to the Texas-Mexican and Mexican-National Railroads; Alice, Tex.


### *CORRESPONDENCE.*

---

### A FEW SURGICAL OBSERVATIONS.

ALICE, TEX., July 2, 1894.

*Editor Atlanta Medical and Surgical Journal:*

The following surgical cases may be of interest to your readers:

CASE 1.—M. de H., age forty-seven, a Spaniard, consulted me in January last about his condition. I found that prior to his seeing me he had had a bubo in both groins, but at that time he suggested that he was suffering from a fistula; so, upon examination I found a subcutaneous sloughing tract which ran from above Poupart's ligament on either side down into the ischio-rectal fossa. Only one or two of the inguinal glands were sloughing, but the others quite indurated, so I extirpated all, and with the aid of my grooved director made an incision from both groins downwards, inwards and backwards into the ischio-rectal fossa, laying the whole tract wide open, and curetting with a sharp spoon. After using a fifteen volume solution of dioxide of hydrogen as an antiseptic bath, and dressings of boric acid and sterilized gauze, the incision (which was twenty-one inches in length) healed by cicatrization in about fifteen days. After three weeks' tonic treatment he was dismissed (seemingly) entirely well.

CASE 2.—Baby S., age two years, boy, stout and apparently in good health, was brought into my office in February last. Examination showed that he had a soft, fluctuating tumor on the outer aspect and slightly above articulation of left knee. No pain on pressure. Family history on both sides tubercular. After putting him on a constitutional treatment of a morrhuol-creosote preparation, I operated and drew off a thick (?) caseous looking fluid, and injected the joint with warm sweet oil and iodoform, as suggested by Professor Pinckney French, of St. Louis. Afterwards I made slight pressure over wound with a flexible rubber cloth bandage, and in two months the limb was entirely well.

Case 3.—T. M., an engineer, consulted me on March 12th about a roaring in his right ear, which had been present for five years. No symptoms of any diagnostic value could be given. Examination revealed a foreign body diagonally across the meatus auditorius externus, which was pressing against the membrana tympani. Injections of sterilized water (borated) failed to move it, but increased its size, causing a great amount of pain. So a pair of Politzer's forceps were applied and the foreign body removed, although with great difficulty, as either end was imbedded in the canal. Inspection showed that it was a well preserved watermelon seed, which patient afterwards said had been "shot" into his ear by a fireman five years previous. Roaring was immediately stopped.

J. D. Westervelt, Jr., M.D.,
Surgeon to the Texas-Mexican and Mexican-National Railroads.

---

### Diagnosis of Early Pregnancy.

Dr. R. L. Dickinson makes an interesting report of original investigations made by him to discover how early pregnancy may be diagnosed. He relies entirely upon the bimanual method of examination. It is easy to see that an expert in this method of examination, and one who has plenty of material, could certainly make a diagnosis at an earlier period than is usually the custom with practitioners. Dr. Dickinson concludes his article as follows: We offer then tentatively six bimanual signs of early pregnancy, stated in the order of their appearance and importance, and in the order of the frequency with which they are found; they run as follows (except that compressibility of the isthmus and the change of the consistency of the body possibly outrank the rest):

1. Bellying or bulging of the body of the uterus.
2. Elasticity or boggyness of the body of the uterus.
3. Compressibility of the lower uterine segment.
4. Transverse fold (about four to six weeks).
5. The longitudinal fold or furrow.
6. The decidual spot (about six to eight weeks).

